# A Nanoscale Design Approach for Enhancing the Li-Ion Conductivity of the Li_10_GeP_2_S_12_ Solid Electrolyte

**DOI:** 10.1021/acsmaterialslett.1c00766

**Published:** 2022-01-26

**Authors:** James A. Dawson, M. Saiful Islam

**Affiliations:** † Chemistry—School of Natural and Environmental Sciences, Newcastle University, Newcastle upon Tyne, NE1 7RU, U.K.; ‡ Centre for Energy, Newcastle University, Newcastle upon Tyne, NE1 7RU, U.K.; § Department of Chemistry, University of Bath, Bath, BA2 7AY, U.K.; ∥ Department of Materials, University of Oxford, Oxford, OX1 3PH, U.K.

## Abstract

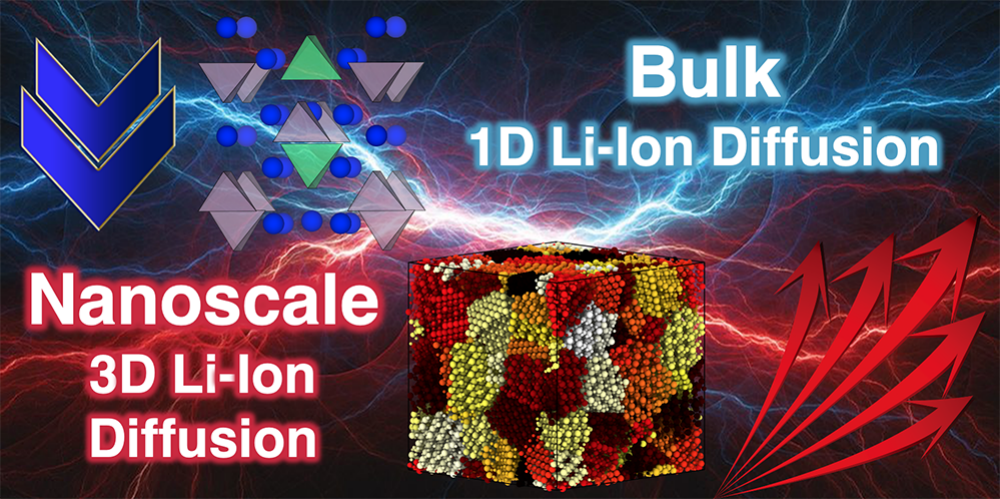

The discovery of the lithium superionic conductor Li_10_GeP_2_S_12_ (LGPS) has led to significant research activity on solid electrolytes for high-performance solid-state batteries. Despite LGPS exhibiting a remarkably high room-temperature Li-ion conductivity, comparable to that of the liquid electrolytes used in current Li-ion batteries, nanoscale effects in this material have not been fully explored. Here, we predict that nanosizing of LGPS can be used to further enhance its Li-ion conductivity. By utilizing state-of-the-art nanoscale modeling techniques, our results reveal significant nanosizing effects with the Li-ion conductivity of LGPS increasing with decreasing particle volume. These features are due to a fundamental change from a primarily one-dimensional Li-ion conduction mechanism to a three-dimensional mechanism and major changes in the local structure. For the smallest nanometric particle size, the Li-ion conductivity at room temperature is three times higher than that of the bulk system. These findings reveal that nanosizing LGPS and related solid electrolytes could be an effective design approach to enhance their Li-ion conductivity.

Solid-state batteries are currently at the forefront of the quest for next-generation energy storage technologies. The development of safe and energy dense solid-state batteries hinges on the replacement of the organic liquid electrolytes found in current commercial batteries with solid electrolytes.^1−5^

Following the first report^6^ of the lithium superionic conductor Li_10_GeP_2_S_12_ (LGPS), this material has attracted considerable attention as a solid electrolyte for solid-state batteries.^7−32^ This interest primarily results from its exceptional room temperature Li-ion conductivity of 12 mS/cm, matching that of current liquid electrolytes.^6^ The LGPS structural family therefore represents one of the crucial components in the development of solid-state batteries.

Nanostructured energy materials have attracted considerable interest because of the potential for unusual properties endowed by confining their dimensions.^33^ However, while the ion transport mechanisms in bulk LGPS have been investigated,^7−9^ this is not the case for nanocrystallite effects in LGPS samples. Grain boundary resistance in sulfide solid electrolytes is generally considered to be minimized compared to that of oxide systems.^34−36^ Beyond LGPS, there are several studies concerning the nanostructure of various solid electrolytes in order to reduce interfacial resistance and improve ion transport.^37−44^ The influence of nanosizing crystalline samples on Li-ion conductivity has been illustrated for sulfide^44−46^ and oxide^47−49^ solid electrolyte materials. However, the atomistic effects of nanosizing LGPS have not been previously characterized.

In this study, we show how reducing the size of LGPS crystallites to the nanoscale results in a substantial enhancement in Li-ion conductivity. Using a novel nanoscale molecular dynamics approach, we are able to directly simulate the Li-ion conductivity of LGPS as a function of its particle size. We find a clear trend of increasing Li-ion conductivity with decreasing particle size resulting from fundamental changes in the ion diffusion pathways and local structures of the nanocrystals. Our findings represent a nanosizing approach for the enhancement of the superionic conductivity of LGPS and have wide implications for the optimization of solid electrolytes in general.

The bulk crystal structure of LGPS is tetragonal (space group *P*4_2_/*nmc*) consisting of negatively charged (PS_4_)^3–^ and (GeS_4_)^4–^ tetrahedra, which are surrounded by Li ions in tetrahedral and octahedral coordination (shown in Figure 1a). The bulk LGPS structure was first simulated with the calculated lattice parameters of *a* = 8.501 Å and *c* = 12.822 Å in good agreement with those obtained experimentally using X-ray diffraction of *a* = 8.718 Å and *c* = 12.635 Å.^6^

**Figure 1:**
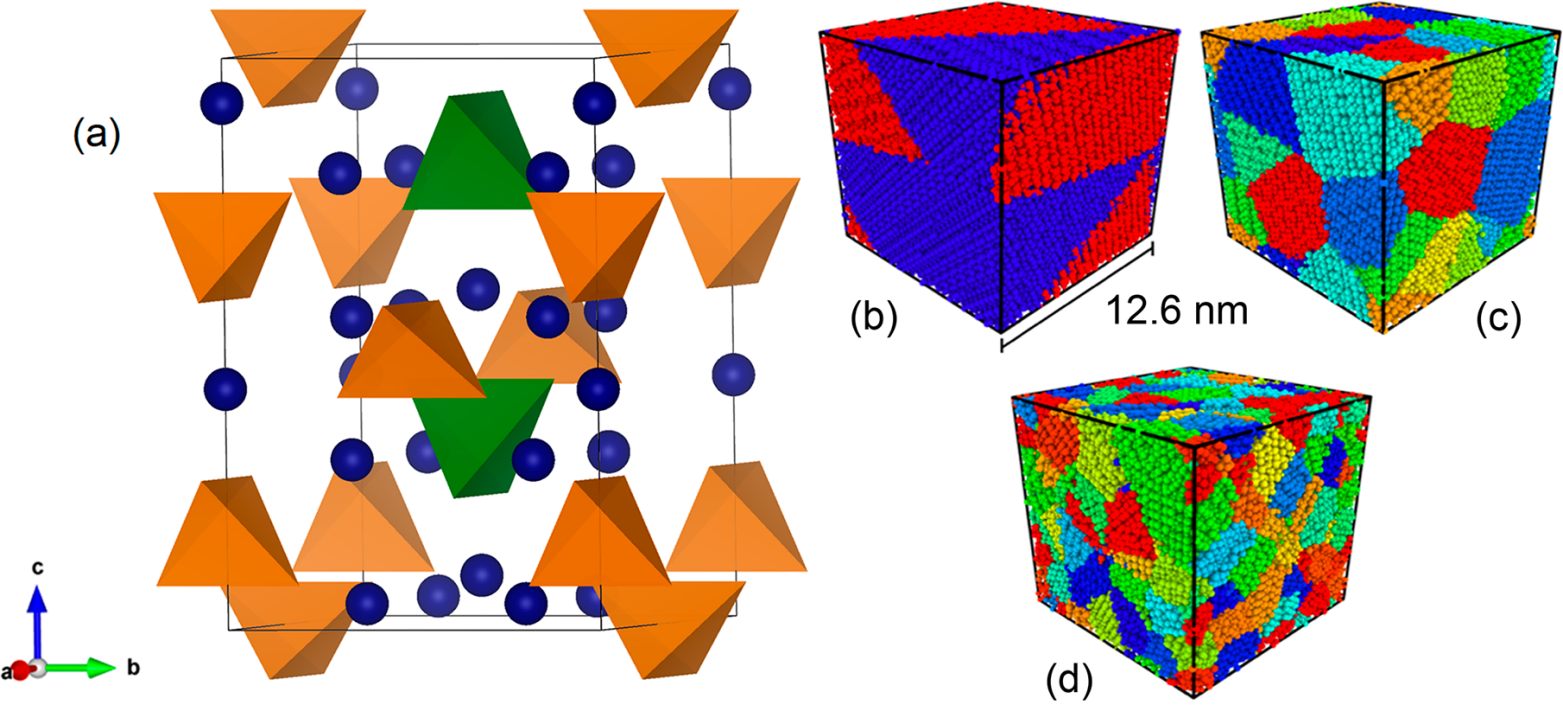
Bulk and nanocrystalline structure of LGPS. (a) Computed bulk Li_10_GeP_2_S_12_ structure taken from the Materials Project^50^ with Li ions in blue and PS_4_ and GeS_4_ tetrahedra in orange and green, respectively. Cubic nanocrystals of LGPS containing (b) two particles with an average volume of 1000 nm^3^, (c) 20 particles with an average volume of 100 nm^3^, and (d) 200 particles with an average volume of 10 nm^3^. The particle volumes are simply determined by dividing the total nanocrystal volume by the total number of particles in that nanocrystal. Each color represents a unique particle.

To determine the influence of the bulk system versus nanosizing on LGPS, we first constructed cubic nanocrystalline systems each consisting of ∼100 000 ions with three different average particle volumes (10, 100, and 1000 nm^3^). These nanocrystals were then investigated using large-scale molecular dynamics (MD) with long simulation times of <10 ns, as detailed in the Experimental Section. We stress that the system sizes and time scales used in this work are orders of magnitude greater than those that can be achieved with ab initio MD.

Examples of the nanocrystals investigated in this study are given in Figure 1b–d. To derive consistent results, three unique nanocrystals were created for each particle volume and the results averaged. The variation between the three nanocrystals for each particle volume is minimal. The process used to construct the nanocrystals is described in the Experimental Section and has been recently applied to successful studies of Na_3_PO_4_ and Na_3_PS_4_ solid electrolytes.^34^

The calculated Li-ion conductivities for bulk and nanocrystalline LGPS are plotted in Figure 2, along with data from experiment.^6,13^ The calculated bulk conductivities are slightly underestimated compared to experimental values for polycrystalline^6,13^ and single-crystal^26^ LGPS but are in good agreement with previous ab initio MD (when extrapolated from high temperatures)^7^ and classical MD simulations.^8^

**Figure 2:**
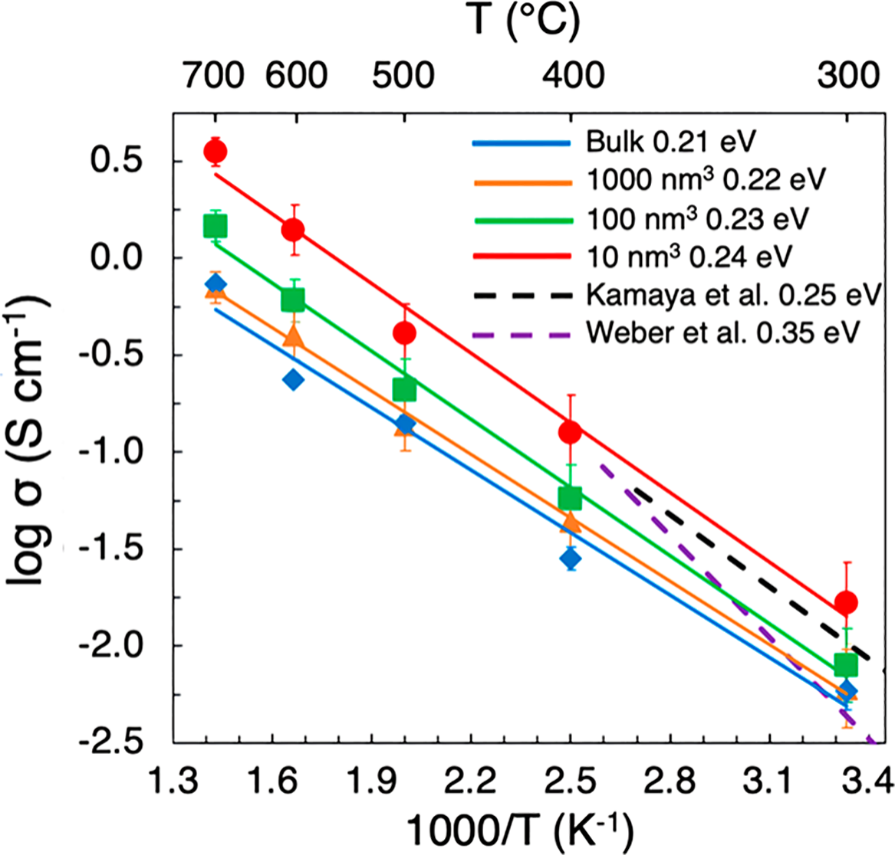
Li-ion transport in bulk and nanocrystalline LGPS. Li-ion conductivities (σ) and activation energies (*E*_a_) for bulk and nanocrystalline LGPS (for three different particle volumes of 1000, 100, and 10 nm^3^) and compared to previous experimental^6,13^ studies.

The results in Figure 2 predict that the Li-ion conductivity of LGPS increases as a result of dramatically decreasing the particle volume, that is, nanosizing. The highest conductivities are found for the smallest particle volume of 10 nm^3^ (with an average particle size of ∼2.15 nm), with a value of 15.10 mS cm^–1^ obtained at 300 K, which is higher than that found in the seminal study of Kamaya et al.^6^ at the same temperature (12 mS cm^–1^) and indeed subsequent experimental studies of LGPS.^13^ Moreover, this conductivity of 15.10 mS cm^–1^ is almost triple the conductivity calculated for the bulk (single crystal) system of 5.87 mS cm^–1^. It is noteworthy that the largest jump in conductivity between the different simulated systems occurs between the smallest particle volumes of 100 and 10 nm^3^.

The calculated activation energies (0.21–0.24 eV) are all in excellent agreement with values derived from impedance (0.22–0.35 eV)^6,10,13,26^ and NMR (0.21–0.26 eV)^10,51^ measurements. In addition to grain boundary resistance often being considered to be minimal in LGPS and its derivatives,^16,18^ our results show that nanocrystalline effects may actually be beneficial to Li-ion conduction in these materials when the particle size is sufficiently small (<10 nm).

Although our results present a clear prediction of the effects of nanosizing on Li-ion transport in LGPS, we recognize that it remains highly challenging to synthesize LGPS particles with the nanoscale average particle sizes (∼2–10 nm) utilized in our simulations. Recent studies^52,53^ have reported the synthesis of LGPS with particle sizes of ∼100–1000 nm; however, these sizes are still substantially larger than those in this study.

Nevertheless, it is encouraging that nanocrystals of the related sulfide electrolyte Li_3.25_P_0.95_S_4_ as small as 5 nm in an amorphous matrix have been reported.^46^ Furthermore, a massive enhancement in Li-ion conductivity was observed for nanoporous Li_3_PS_4_ with average particle sizes of 80–100 nm,^44^ although there is debate that such nanoporous samples may in fact be a mixture of several materials.^54^ Recently, the utilization of ultimate-energy mechanical alloying and rapid thermal annealing to produce nanosized Li-argyrodite solid electrolyte particles of ∼20 nm (and an anonymously high Li-ion conductivity as a result) is highly encouraging.^55^ In addition, it is important to bear in mind that sizes of <10 nm^3^ are difficult to detect using XRD and instead appear as amorphous.^46,56^ Therefore, it may be necessary to utilize other techniques, such as transmission electron microscopy, to observe such minute crystalline domains. Additional discussion regarding the stability and synthesizability of such nanocrystalline materials is provided in the Supporting Information.

Given the similarities between their structural and ion transport properties, it is important to emphasize that the enhancement in ion conductivity found for LGPS as a result of nanosizing may also be applicable to many of its derivatives; these include Li_9.54_Si_1.74_P_1.44_S_11.7_Cl_0.3_,^17^ an even faster Li-ion diffusing solid electrolyte, which warrants future investigation.

It is known that the dimensionality of Li-ion transport within crystal structures of solid electrolyte materials is important for their ionic conductivity. The impact of the disorder caused by nanocrystals on the Li-ion transport in LGPS can be observed by visualizing the Li-ion trajectories during the simulations. Figure 3a shows the accumulated Li-ion trajectories for bulk LGPS at 300 K. As reported in previous experimental and computational studies,^6−8,13,26,57^ the most facile Li-ion diffusion occurs in one-dimensional channels along the *c* direction of the tetragonal structure, with diffusion also taking place in the *ab* plane. This is also supported by Figure 3c, where the mean squared displacement (MSD) of Li ions is plotted for the three primary directions. We find that diffusion in the *c* direction is 5-fold higher than in the *ab* plane. Such anisotropic conductivity is also observed from impedance measurements of LGPS single crystals.^26^

**Figure 3:**
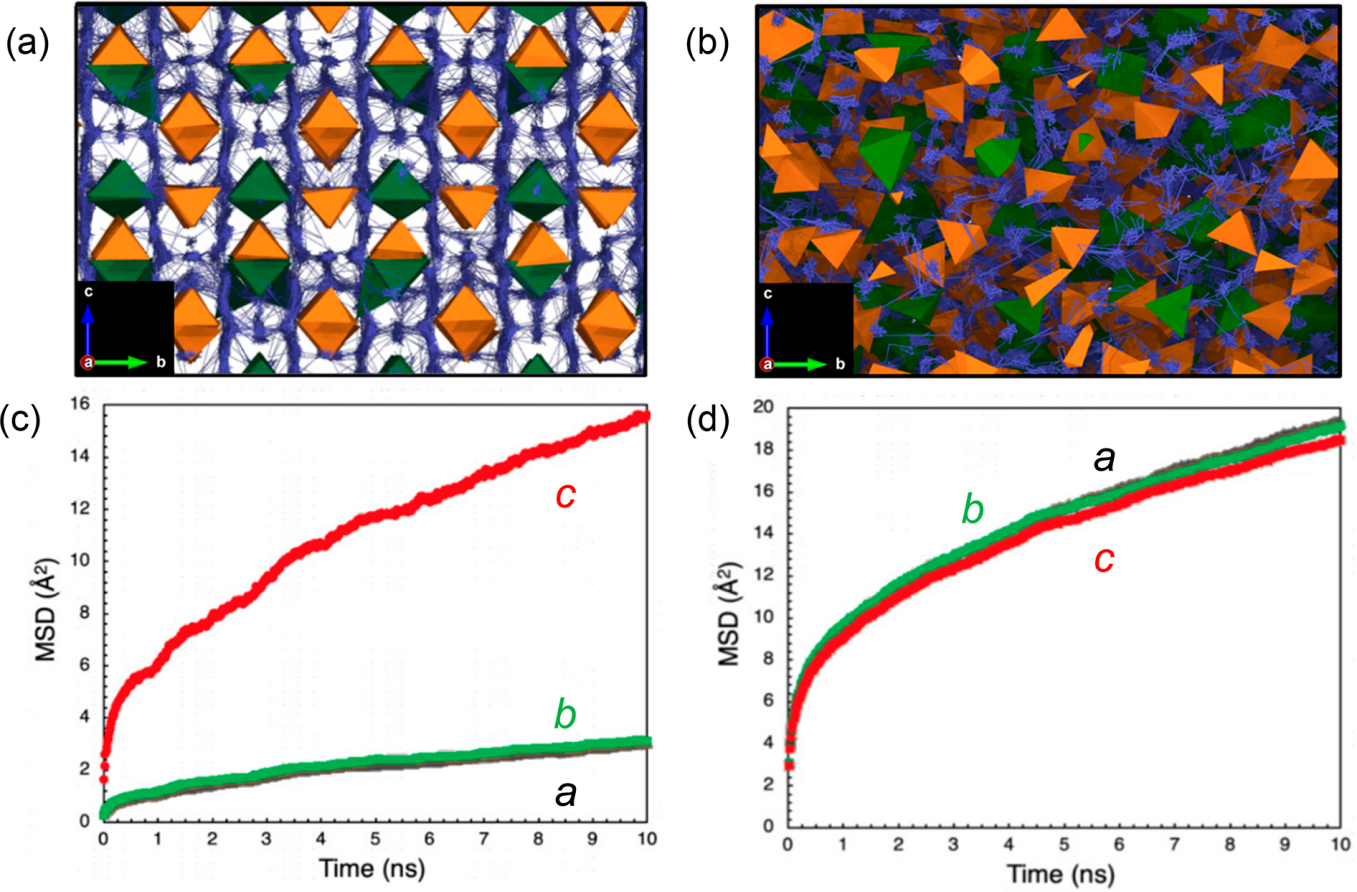
Li-ion diffusion pathways in bulk and nanocrystalline LGPS. Diffusion density plots of Li ions (blue) overlaid on GeS_4_ (green) and PS_4_ (orange) tetrahedra in (a) bulk and (b) nanocrystalline LGPS with a particle volume of 10 nm^3^ at 300 K (2.4 × 3.5 nm cross section). MSD plots of Li-ion diffusion in (c) bulk and (d) nanocrystalline LGPS with a particle volume of 10 nm^3^ at 300 K for the *a*, *b*, and *c* directions.

Figure 3b presents the Li-ion trajectories for nanocrystalline LGPS with the smallest particle volume of 10 nm^3^ at 300 K. In contrast to the well-defined Li-ion diffusion pathways for bulk LGPS, the diffusion pathways for nanosized LGPS are far more isotropic, indicating fast diffusion in all directions. This is further corroborated by the MSD plots given in Figure 3d, where it is now clear that there is no longer a preference for Li-ion diffusion in the *c* direction, with a significant increase in the in-plane diffusion resulting in 3D Li-ion diffusion.

The additional advantage of nanosizing is the short path length for Li-ion transport and the increased intergranular diffusion, as found in Figure 3b, which increases with decreasing particle size. Hence, the decreased particle size in the nanocrystals dominates the contribution to the Li-ion conductivity and allow the Li ions to readily diffuse in all directions, beyond the preferential diffusion pathways in the *c* and *ab* directions in bulk LGPS. Such isotropic 3D conduction behavior allows lithium access through all surfaces of the LGPS particles. High surface areas of solid electrolyte particles may also allow greater contact areas with the electrodes, and hence a high Li-ion flux across the interface.^33^ Furthermore, related findings have been reported for the nanoporous form of a parent material of LGPS, Li_3_PS_4_, where the ionic conductivity has a positive correlation with the surface area of the material due to the presence of microstrain and high concentrations of defects at the surface.^44^

To investigate and understand the local structural factors that influence Li-ion transport in these nanocrystalline systems, we analyze the radial distribution functions (RDFs) of ion pairs in LGPS. Figure 4 shows a comparison of the RDFs for the Li–Li, Li–S, Ge–Ge, and P–P pairs of the bulk versus nanocrystalline system (for the smallest particle volume of 10 nm^3^). Two key features emerge. First, the values beyond the first maximum peak for Li–Li indicate that the Li distribution is disordered in LGPS indicative of a superionic conductor. We note that the main maximum peaks of the bulk RDFs at 2.36 and 3.40 Å for Li–S and Li–Li, respectively, are in excellent agreement with the experimental structure,^6^ as well as RDFs calculated using ab initio MD.^58^

Second, all the RDFs for the nanocrystals exhibit broader peaks especially after the first maximum peak and are associated with the increased disorder in these systems. By far the greatest difference in the nanocrystal compared to the bulk is found for the Ge–Ge and P–P RDFs, with significant peak broadening in the nanocrystal. This increased disorder agrees with the distinct differences found in the diffusion density plots and ion conduction pathways shown in Figure 3a and b.

**Figure 4:**
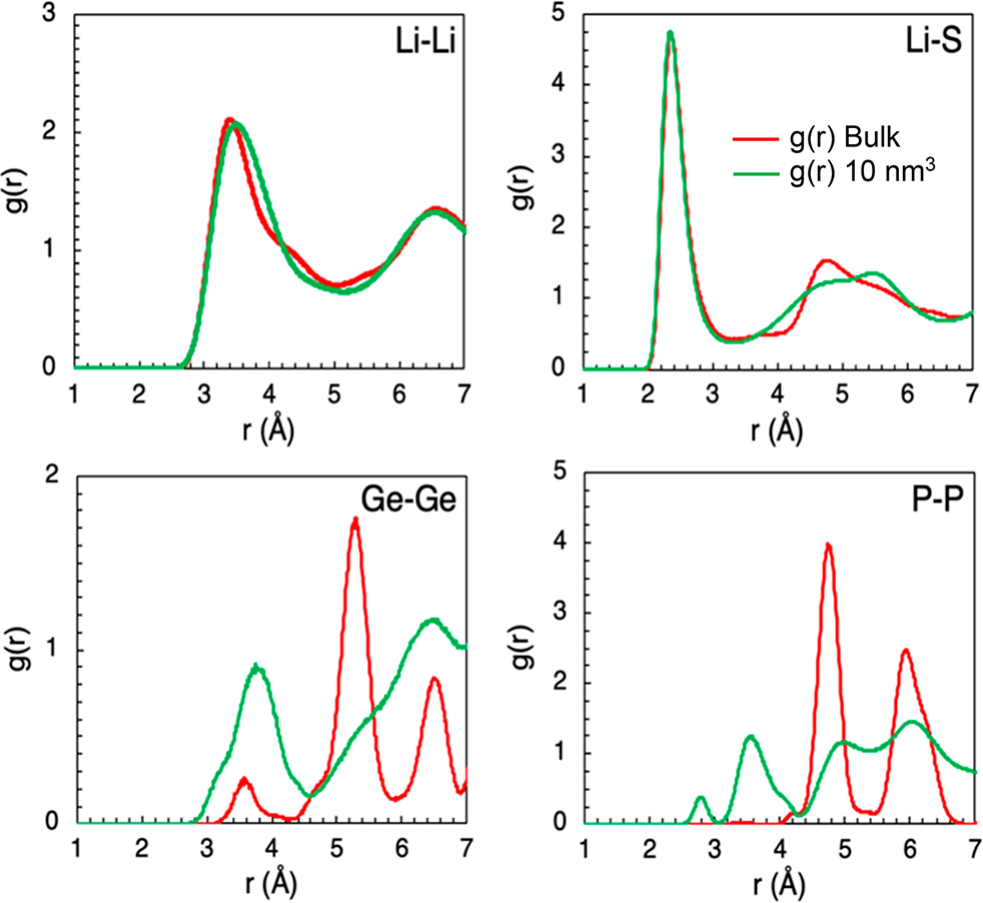
Local structural differences between bulk and nanocrystalline LGPS. Radial distribution functions (RDFs) of Li–Li, Li–S, Ge–Ge, and P–P RDFs (*g*(*r*)) for bulk (red) and nanocrystalline (green, particle volume of 10 nm^3^) LGPS at 300 K.

The remaining RDFs for bulk LGPS and the smallest particle size (10 nm^3^) are given in Figure S1. It is noteworthy that the cation-S coordination for all tetrahedra (PS_4_, GeS_4_ and LiS_4_) and octahedra (LiS_6_) are similar at short distances (<3 Å) between the nanocrystals and the bulk. This means that the fundamental tetrahedra and octahedra in the material are unchanged during nanosizing and that it is cation–cation disorder and Li–S disorder at >4.5 Å that are particularly important in the change in conduction mechanism and increased Li-ion conductivity (indicated in Figure 3). The RDFs for the larger particle size systems (100 and 1000 nm^3^) also show similar differences to the bulk RDFs as the 10 nm^3^ system but to a weaker extent and are therefore not presented.

To further illustrate the influence of ion coordination on the Li-ion transport of nanosized LGPS, we plot the difference in the Li–Li and Li–S coordination numbers between bulk (*n*(*r*)_bulk_) and nanocrystalline (10 nm^3^, *n*(*r*)_nano_) LGPS, shown in Figure 5. In these plots, values above zero reflect higher coordination in the nanocrystal compared to the bulk, while values below zero represent reduced coordination in the nanocrystal.

**Figure 5:**
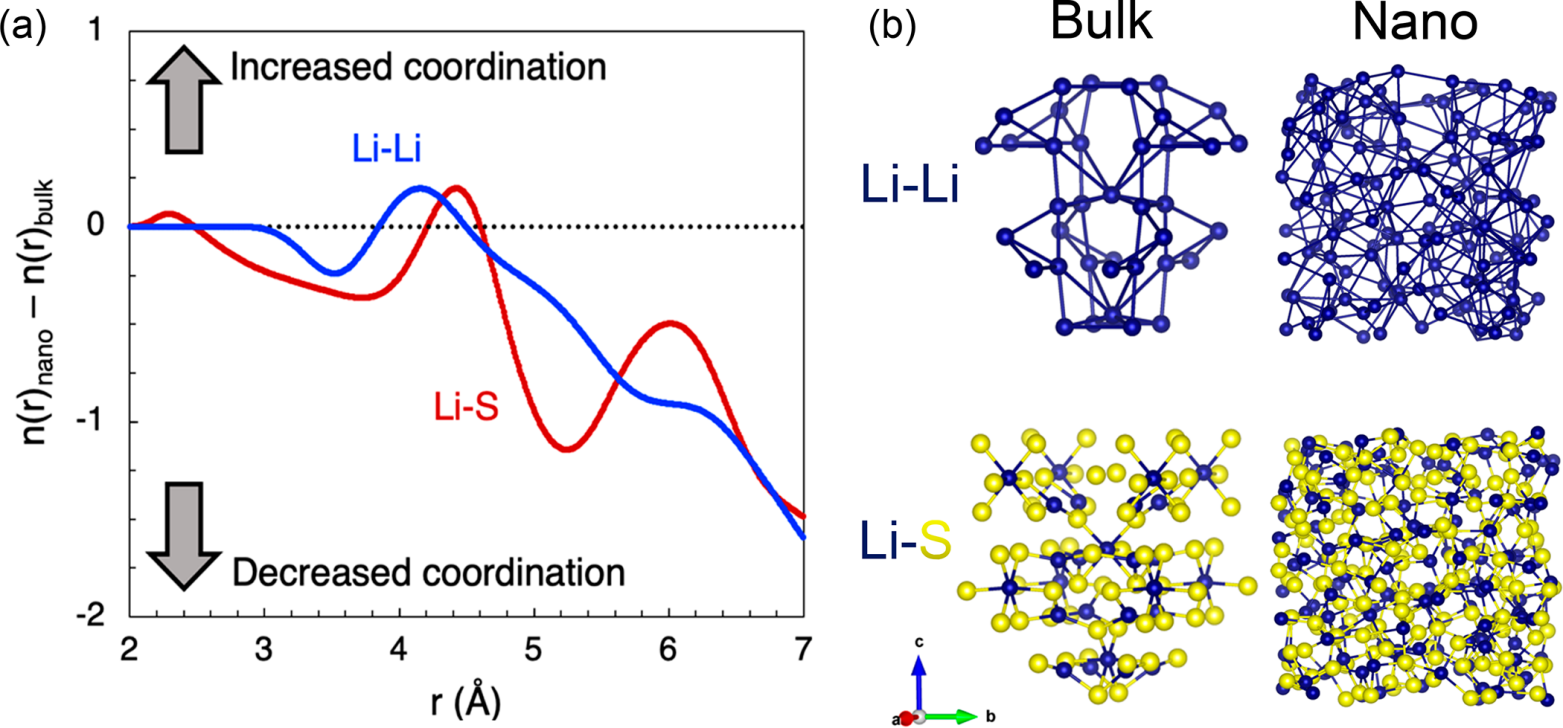
Ion coordination differences between nanocrystalline and bulk LGPS. (a) Plots of difference between Li–Li and Li–S coordination numbers in nanocrystalline (10 nm^3^, *n*(*r*)_nano_) and bulk (*n*(*r*)_bulk_) LGPS as a function of r at 300 K. Positive and negative values represent increased and decreased coordination, respectively. (b) Schematics of Li–Li and Li–S substructures in bulk LGPS (left) and a 0.2 × 0.2 × 0.2 Å^3^ section of a 10 nm^3^ LGPS nanocrystal (right).

The results in Figure 5 show that Li–Li and Li–S coordination decreases by one to two for distances of >4.6 Å. Intuitively, this decrease in Li–S coordination is likely to contribute to the high Li-ion conductivity observed in the nanocrystalline systems. In contrast, the role of reduced Li–Li coordination is not as clear. On the basis of transition state theory and the computational screening of solid Li-ion conductors,^59^ higher Li–Li coordination enhances Li-ion conductivity by providing more accessible Li sites. Therefore, whether it is the increased Li–Li coordination at ∼4 Å or the decreased Li–Li coordination beyond >4.6 Å that plays the most significant role in enhancing the Li-ion conduction in the nanocrystals needs further investigation.

Regardless, these changes in local structure resulting from the nanosizing of LGPS must be major factors in the enhancement of Li-ion conductivity in this material, given that the lithium concentration is similar for each nanocrystalline system (Table S3) and that no extrinsic dopants have been added.

We, reported, similar findings in a previous study,^34^ where the decrease in Na coordination (undercoordination) resulted in an enhancement in Na-ion transport for polycrystalline Na_3_PS_4_. However, it is noteworthy that the local structural changes in LGPS in this work are more substantial than those found for Na_3_PS_4_. It is also important to consider that for the nanocrystals with the smallest size considered in this work, the local structures are likely to be highly affected by surface effects, as has been shown for Li_3_PS_4_.^44^ We note that it is beyond the focus of this simulation study to include conductivity measurements, and indeed one of our aims is to stimulate further experimental work in this area. Furthermore, it is currently unknown how the nanosizing approach presented, here, will affect the stability and interfacial resistance of LGPS when in contact with the electrodes in a solid-state battery.

In conclusion, despite the high Li-ion conductivity in the promising solid electrolyte material, Li_10_GeP_2_S_12_, the effects of nanosizing have not been fully investigated. Through a nanoscale simulation approach, we have demonstrated that moving from bulk LGPS to the nanoscale results in a large enhancement in Li-ion conductivity; our atomistic analysis indicates that this effect results from the combination of significant changes in both the ion conduction pathways and the local structural environment. In bulk LGPS, Li-ion transport is anisotropic with faster diffusion in the *c* direction than within the *ab* plane. In contrast, for LGPS particles with nanometric sizes, there is a switch to isotropic 3D Li-ion conduction with fast transport in all directions. We also find greater local structural cation–cation disorder and a decrease in Li–S coordination (or undercoordination) with decreasing particle volume, which also facilitates the high Li-ion conductivity.

This study confirms that control of dimensions on the nanoscale can have a profound influence on performance. Despite the challenges of nanoscale synthesis, these findings open up an alternative nanosizing approach to enhance the Li-ion conductivity of LGPS and suggests an important design route that has potential to be widely relevant to ion conductors in general.

## Experimental Section

The MD simulations in this work are based on established techniques and have been widely used to determine the ion transport properties in a wide variety of Li- and Na-ion solid electrolytes.^60−63^ The calculations were performed using the LAMMPS code^64^ with long MD runs of 10 ns were completed using a time step of 1 fs and supercells of ∼100 000 ions for both the bulk (single crystal) and nanocrystal systems. The bulk crystal structure of LGPS was obtained from the Materials Project,^50^ with the disordered site occupancies ordered using the same method used in previous studies.^65^

Simulations were carried out for a temperature range of 300–700 K at intervals of 100 K using the NVT ensemble with a Nose–Hoover thermostat,^66^ with initial equilibration performed using the NPT ensemble for ∼2 ns. Self-diffusion data for Li were obtained from an MSD analysis according to
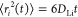
1where  is the MSD, *D*_Li_ is the diffusion coefficient for Li and *t* is time. The diffusion data were then converted to conductivities (σ) using the Nernst–Einstein relationship:
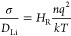
2where *n* is the number of Li ions per unit volume, *q* is the electron charge, *k* is the Boltzmann constant, *T* is the temperature, and *H*_R_ is the Haven ratio, which is set to three in our calculations based on the ab initio MD simulations of He et al.^65^

The potential model of Kim et al.^61^ developed for (Li_2_S)_0.75_(P_2_S_5_)_0.25_ and Li_3_PS_4_ was used for the simulations in this work, with the addition of a newly derived Morse potential for the Ge–S interaction in LGPS (using the GULP code^67^). This potential was successfully used to study Li-ion transport in both crystalline and glassy thiosulfate solid electrolytes,^61^ thereby illustrating its ability to deal with significantly disordered systems, such as those featured in this work. The model was fit to a variety of structural and thermomechanical parameters, including the lattice parameters and shear, bulk, and elastic moduli, from both experiment and density functional theory (DFT). The atomic charge of each species was determined using Bader analysis.^68^

The additional Ge–S interaction was then fit based on the LGPS structure and its charge was also determined using Bader analysis based on DFT with the VASP code.^69^ The projector augmented wave method^70^ and the PBEsol exchange-correlation functional^71^ were employed with a plane-wave cutoff energy of 520 eV and a k-point mesh spacing smaller than 0.05 Å^–1^. The full potential model and atomic charges are tabulated in Table S1. The calculated lattice parameters for LGPS of *a* = 8.501 Å and *c* = 12.822 Å are in good agreement with those obtained using X-ray diffraction of *a* = 8.718 Å and *c* = 12.635 Å.^6^

To confirm that the main findings of the study were not simply an artifact of this model, an additional LGPS potential model was developed from ab initio molecular dynamics data. The design of this model is described in the Supporting Information and its parameters are provided in Table S2. This new model was used to test the reliability of the existing model by repeating the MD simulations on the same bulk and nanocrystalline LGPS systems with the exact same computational parameters described above. An Arrhenius plot of the conductivities calculated using the new model is given in Figure S2 along with the data from the existing model (Figure 2). It is clear that the same trend of increasing Li-ion conductivity with decreasing particle size is maintained in the results from the new model. Therefore, both models, although developed independently of each other using completely different methods, confirm the primary findings of our study. The calculated room-temperature conductivities and activation energies are also similar between the two models.

The nanocrystal models used in this study were constructed using Voronoi tessellations, as employed in the Atomsk program,^72^ in which nodes are introduced at given positions inside the simulation box that are then linked with their neighboring nodes. The normals to these links are then found and these define the contours of the randomly orientated particles, that is, the particle boundaries in this study. Unit cells are then placed at the nodes and are expanded in three dimensions. The final nanocrystal is then obtained after the unit cells have been expanded and cut into the respective particles.

Cubic nanocrystals with dimensions of 126 × 126 × 126 Å^3^ were used. Nanocrystals with 2, 20, and 200 particles (equivalent to particle volumes of 1000, 100, and 10 nm^3^, respectively) were used to investigate the effects of nanosizing on Li-ion transport in LGPS. MD simulations were carried out on three different random polycrystals for each particle volume and the data were averaged. The differences between the average stoichiometries of the nanocrystals are minimal and do not have a significant influence on the presented results, as shown in Table S3. As can be seen from their stoichiometries, the nanocrystals have small vacancy concentrations, which is commensurate with the fact that experimental samples with such small particle sizes will have reduced density. Nevertheless, these vacancies do not explain the observed trend of increasing Li-ion conductivity with decreasing particle size.

The new method presented here is superior to the analysis of single particle boundaries for analyzing ion transport since it accounts for hundreds of particle boundaries simultaneously as found in a real material and allows us to consider conductivity as a function of particle size. While the analysis and simulation of single particle boundaries is certainly simpler, there is no guarantee that the Li-ion conduction mechanisms of these particle boundaries would be representative of a true sample.

## Supporting Information Available

Potential model parameters used, the average stoichiometries for the LGPS nanocrystals, details regarding the stability and synthesizability of the nanocrystalline models, and additional RDFs (PDF)
