# Supplementary material for: A Nanoscale Design Approach for Enhancing the Li-Ion Conductivity of the Li10GeP2S12 Solid Electrolyte
Source: ACS Mater Lett. 2022 Jan 26;4(2):424–31. doi: 10.1021/acsmaterialslett.1c00766 (PMC9097573; doi:10.1021/acsmaterialslett.1c00766)
Supplement: Supplementary file 1 — tz1c00766_si_001.pdf [file tz1c00766_si_001.pdf]

Supporting Information for

A Nanoscale Design Approach for Enhancing the Li-Ion Conductivity of the $Li_{10}GeP_2S_{12}$ Solid Electrolyte

James A. Dawson*[,a,b] and M. Saiful Islam*[,c,d]

[a]Chemistry – School of Natural and Environmental Sciences, Newcastle University, Newcastle upon Tyne, NE1 7RU, UK

[b]Centre for Energy, Newcastle University, Newcastle upon Tyne, NE1 7RU, UK

[c]Department of Chemistry, University of Bath, Bath, BA2 7AY, UK

[d]Department of Materials, University of Oxford, Oxford, OX1 3PH, UK

*Corresponding authors. Emails: james.dawson@newcastle.ac.uk; saiful.islam@materials.ox.ac.uk

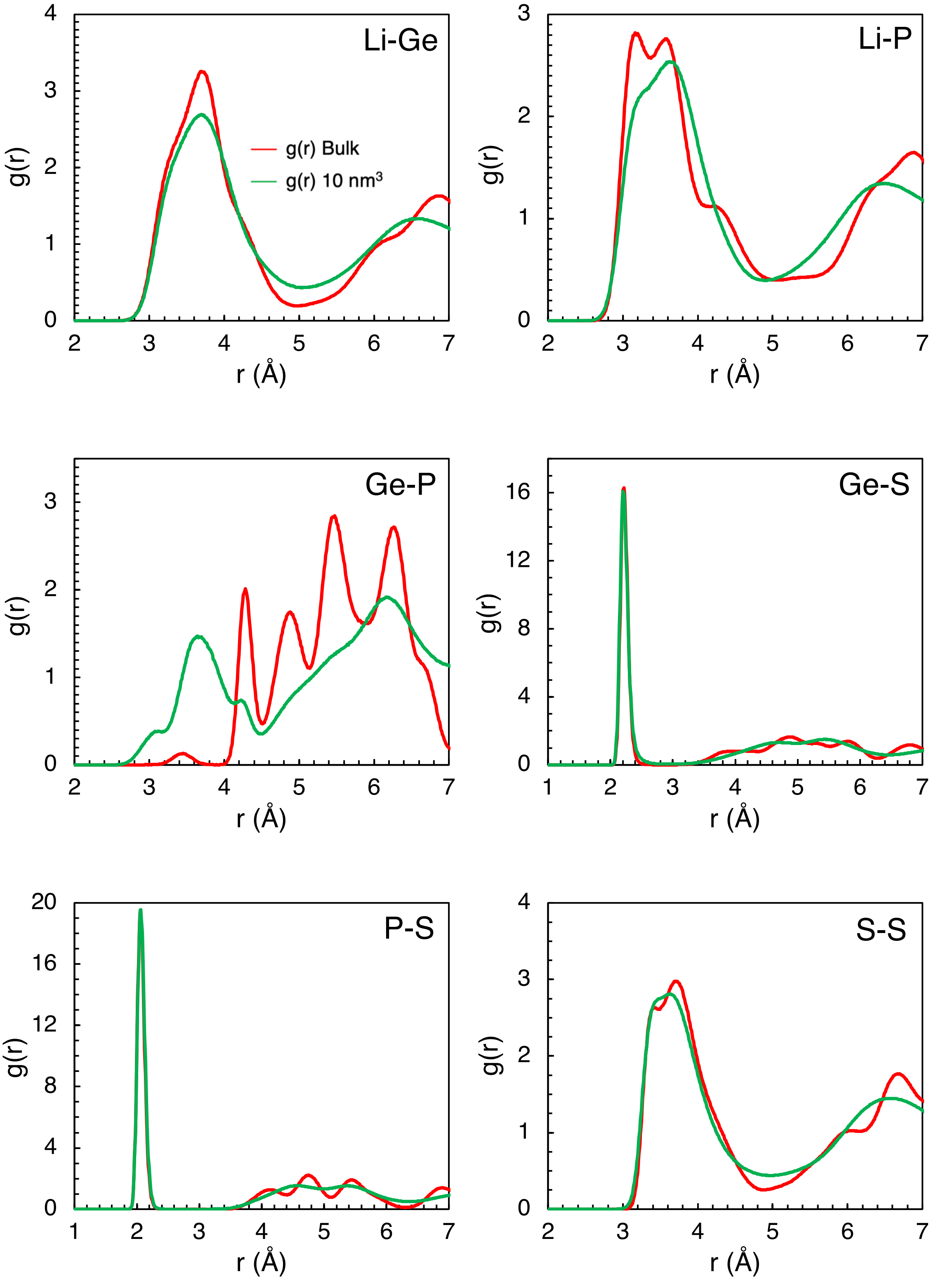

Figure S1. Li-Ge, Li-P, Ge-P, Ge-S, P-S and S-S RDFs (g(r)) for bulk and nanocrystalline (particle volume of 10 nm^3) LGPS at 300 K.

Table S1. Interatomic Morse potential parameters (*D* is the well depth, α is the stiffness parameter and r_0 is the equilibrium distance between the ions) for LGPS taken from Kim et al.,[1] with the exception of Ge-S, which was derived in this study.

Interaction	*D* (eV)	α (Å^{-1})	r_0 (Å)
$Li^{0.8803+}$–$Li^{0.8803+}$	0.0580	3.987	3.404
$Li^{0.8803+}$–$S^{1.048-}$	0.0408	1.399	3.204
$Ge^{1.257+}$–$S^{1.048-}$	0.3147	2.257	2.409
$P^{1.258+}$–$S^{1.048-}$	0.4104	2.329	2.200
$S^{1.048-}$–$S^{1.048-}$	0.0241	1.359	4.284

Development of alternative potential model

To confirm that the main findings of the study were not simply an artefact of the above model, we developed an entirely new LGPS potential model built completely from *ab initio* molecular dynamics trajectories. This model was built using the recently developed POtential Parameter Optimisation for Force-Fields (PopOff) code.[2] PopOff is a Python module that fits Coulomb-Buckingham-type interatomic potentials for classical potential-based molecular dynamics. It is a modular fitting code that allows for increased control over several important aspects of the potential. PopOff fits to first principles derived forces and stress tensors obtained from a VASP training set. The new Buckingham potential parameters are given in Table S2.

Table S2. Interatomic Buckingham potential parameters (*A* represents the size of the repulsive wall, ρ is the length of the repulsive interaction and *C* is the size of the attractive dispersion term) for LGPS developed using PopOff.

Interaction	*A* (eV)	ρ (Å)	*C* (eV Å^6)
$Li^{0.8803+}$–$S^{1.048-}$	908.4390	0.3300	45.3748
$Ge^{1.257+}$–$S^{1.048-}$	16658.1377	0.2121	55.5205
$P^{1.258+}$–$S^{1.048-}$	16882.5873	0.1841	0.5295
$S^{1.048-}$–$S^{1.048-}$	26919.0143	0.1578	22.1388

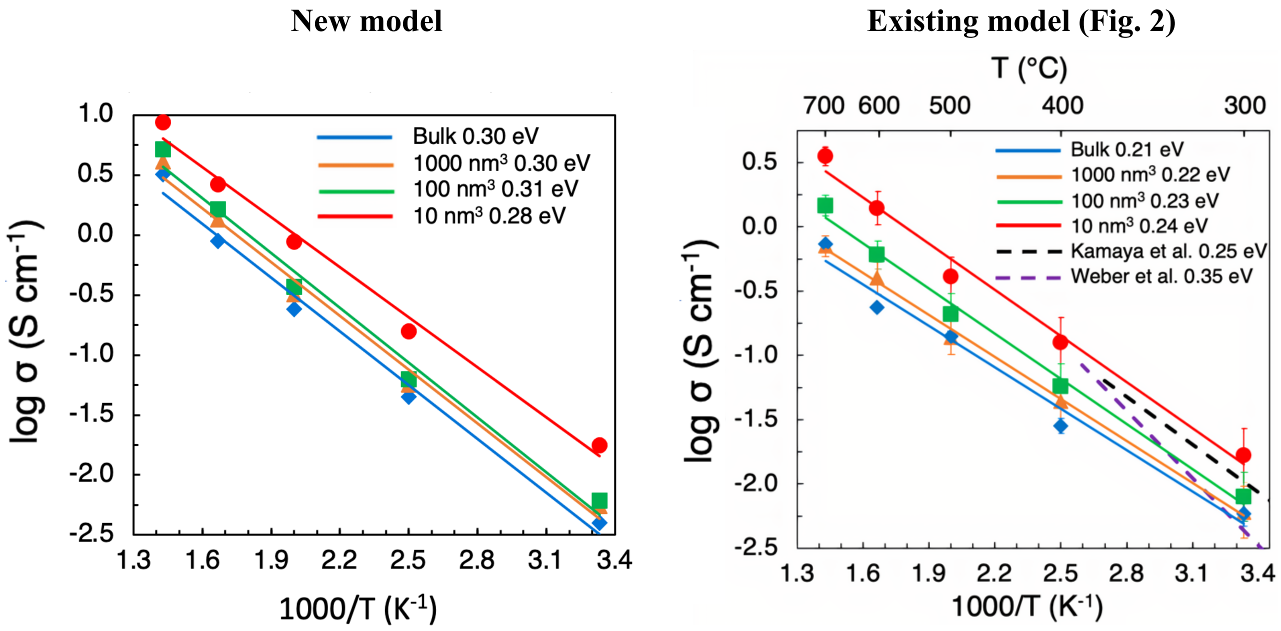

Figure S2. Comparison of calculated Li-ion transport in bulk and nanocrystalline LGPS using the two potential models.

Table S3. Average stoichiometries for LGPS nanocrystals.

Particle volume (nm^3)	Average stoichiometry
1000	$Li_{9.65}Ge_{0.99}P_{1.92}S_{11.61}$
100	$Li_{9.61}Ge_{0.96}P_{1.92}S_{11.53}$
10	$Li_{9.61}Ge_{0.95}P_{1.92}S_{11.51}$

Stability and synthesizability of nanocrystalline models

The smallest systems (particle volume of 10 nm^3) inherently contain higher concentrations of particle boundaries and levels of disorder and are therefore less enthalpically favorable than larger particle size system. For example, the average enthalpy of the 10 nm^3 LGPS systems is 0.015 eV/ion lower than for the average enthalpy of the 1000 nm^3 LGPS systems. Nevertheless, this is true of any polycrystalline system and it does not account for favorable entropic effects. It is certainly a significant challenge to reach the particle volumes considered in this study experimentally but the recent utilization of ultimate-energy mechanical alloying and rapid

thermal annealing to produce Li-argyrodite solid electrolyte particles of ~20 nm (and an anonymously high Li-ion conductivity as a result) is highly encouraging.[3]

References

(1) Kim, J.-S.; Jung, W. D.; Son, J.-W.; Lee, J.-H.; Kim, B.-K.; Chung, K.-Y.; Jung, H.-G.; Kim, H. *ACS Appl. Mater. Inter.* **2019**, *11*, 13–18.

(2) https://github.com/LMMorgan/PopOff

(3) Jung, W. D.; Kim, J.-S.; Choi, S.; Kim, S.; Jeon, M.; Jung, H.-G.; Chung, K. Y.; Lee, J.-H.; Kim, B.-K.; Lee, J.-H.; Kim, H. *Nano. Lett.* **2020**, *20*, 2303–2309.
